# Distinct Roles of Outer Membrane Porins in Antibiotic Resistance and Membrane Integrity in *Escherichia coli*

**DOI:** 10.3389/fmicb.2019.00953

**Published:** 2019-04-30

**Authors:** Umji Choi, Chang-Ro Lee

**Affiliations:** Department of Biological Sciences and Bioinformatics, Myongji University, Yongin, South Korea

**Keywords:** antibiotic resistance, porins, membrane integrity, OmpA, OmpC, OmpF

## Abstract

A defining characteristic of Gram-negative bacteria is the presence of an outer membrane, which functions as an additional barrier inhibiting the penetration of toxic chemicals, such as antibiotics. Porins are outer membrane proteins associated with the modulation of cellular permeability and antibiotic resistance. Although there are numerous studies regarding porins, a systematic approach about the roles of porins in bacterial physiology and antibiotic resistance does not exist yet. In this study, we constructed mutants of all porins in *Escherichia coli* and examined the effect of porins on antibiotic resistance and membrane integrity. The OmpF-defective mutant was resistant to several antibiotics including β-lactams, suggesting that OmpF functions as the main route of outer membrane penetration for many antibiotics. In contrast, OmpA was strongly associated with the maintenance of membrane integrity, which resulted in the increased susceptibility of the *ompA* mutant to many antibiotics. Notably, OmpC was involved in both the roles. Additionally, our systematic analyses revealed that other porins were not involved in the maintenance of membrane integrity, but several porins played a major or minor role in the outer membrane penetration for a few antibiotics. Collectively, these results show that each porin plays a distinct role in antibiotic resistance and membrane integrity, which could improve our understanding of the physiological function and clinical importance of porins.

## Introduction

The outer membrane (OM) of Gram-negative bacteria is a unique architecture which is composed of phospholipids, lipopolysaccharides (LPS), lipoproteins, and β-barrel porins (Henderson et al., 2016). The OM functions as an additional barrier for blocking the transport of toxic compounds, such as bile acid and antibiotics (O’Shea and Moser, 2008; Pages et al., 2008). Therefore, chemicals with a molecular weight of more than 600 Da cannot generally penetrate the envelope of the Gram-negative bacteria (O’Shea and Moser, 2008). Therefore, antibiotics with a molecular weight more than 1400 Da, such as vancomycin and daptomycin, cannot pass through the OM of Gram-negative bacteria. This feature of the OM is one of the main obstacles in the development of a novel antimicrobial agent targeting the Gram-negative pathogens (Lee et al., 2013).

OM porins are transmembrane pore-forming proteins with a β-barrel structure, which forms a water-filled open channel and allows the passive transport of hydrophilic compounds (Schulz, 2002; Nikaido, 2003; Pages et al., 2008). The porins are the most abundant proteins of the OM in Gram-negative bacteria, with various existing types. They can be classified as non-specific or specific porins based on their activity. Additionally, they are classified into monomeric, dimeric, or trimeric porins based on their functional structure (Koebnik et al., 2000; Pages et al., 2008). In addition to the passive transport of various molecules, porins seem to play an important role in maintaining the envelope integrity of the Gram-negative bacteria. For example, outer membrane protein A (OmpA) is a non-specific porin which allows the passive transport of many small chemicals (Sugawara and Nikaido, 1992; Iyer et al., 2018). It is also a peptidoglycan-associated protein with a flexible periplasmic domain that is involved in the non-covalent interaction with peptidoglycan (Samsudin et al., 2016). Because porins mediate the passive diffusion of antibiotics across the OM, they are closely associated with antibiotic resistance in the Gram-negative bacteria. For example, β-lactams and fluoroquinolones were known to penetrate the OM through the non-specific porin OmpF (Mach et al., 2008; Delcour, 2009). Therefore, the *ompF* mutant was resistant to several β-lactam antibiotics in some Gram-negative pathogens, including *Escherichia coli* (Nikaido et al., 1983; Ziervogel and Roux, 2013), *Klebsiella pneumoniae* (Sugawara et al., 2016), *Serratia marcescens* (Moya-Torres et al., 2014), *Pseudomonas aeruginosa* (Okamoto et al., 2001), and *Enterobacter aerogenes* (Bornet et al., 2000). On the other hand, the deletion of OmpA resulted in increased susceptibility to several antibiotics including β-lactams in *Acinetobacter baumannii* (Smani et al., 2014). This result may be caused by the effect of OmpA on the maintenance of membrane integrity, because impaired membrane integrity can increase the intracellular diffusion of antibiotics. Although these results suggest that porins affect antibiotic resistance in different ways, a systematic analysis of the relationship between porins and antibiotic resistance has not been performed yet. In particular, the role of specific porins in antibiotic resistance remains unclear.

In this study, we analyzed how all porins of *E. coli* affect the resistance to various antibiotics of different classes, and the maintenance of membrane integrity. These analyses showed that porins can be classified into three groups according to their roles in antibiotic transport and membrane integrity: antibiotic transport-related specific porins (LamB, YddB, etc.), membrane integrity-related non-specific porin (OmpA), and non-specific porins involved in both antibiotic transport and membrane integrity (OmpC and OmpF). OmpF and OmpA play a major role in antibiotic transport and the maintenance of membrane integrity, respectively. OmpC is important for both of the two functions. These results suggest the functional diversity of porins and explain why the effect of porins on antibiotic resistance is diverse depending on the kind of porin.

## Materials and Methods

### Bacterial Strains, Plasmids, and Culture Conditions

The bacterial strains and plasmids used in this study are listed in Supplementary Table S1. Bacterial cells were grown as described previously (Lee et al., 2015; Choi et al., 2016). Unless otherwise indicated, Luria-Bertani broth (LB broth) was used for the general growth of cells. The antibiotics kanamycin (Kan) and chloramphenicol (Cm) were used at concentrations of 50 μg/ml and 5 μg/ml, respectively. All porin deletion mutants were constructed by replacing the entire or partial open reading frame region of a target gene with the kanamycin-resistance gene using the λ red recombinase as described previously (Datsenko and Wanner, 2000). The kanamycin-resistance gene was amplified from pKD13 using the primer sets listed in Supplementary Table S2. The purified PCR product was electroporated into MG1655 cells harboring a pKD46 plasmid, and the deletion mutant was selected on LB plates with kanamycin at 37°C. The kanamycin-resistance gene was removed by using a pCP20 plasmid expressing the FLP recombinase as described previously (Datsenko and Wanner, 2000).

The *ompA*ΔC mutant was constructed by replacing theregion between 197th codon and the stop codon of the *ompA* gene with the kanamycin-resistance gene containing its promoter region. The kanamycin-resistance gene was amplified using a forward primer with a synthetic linker (underlined) and FLAG-tag (in boldface type), 5′-ACGGCATGCTGAGCCTGGGTGTTTCCTACCGTTTCGGTC AGGGCGAAGCAGGCAGCGGC **GACTACAAAGACGATGAC** **GACAAG**TAGCTTAGACGTCAGGTGGCACT-3′ and a reverse primer, 5′-AAAGGCAAAAAAAACCCCGCAGCAGCGGG GTTTTTCTACCAGACGAGAACACGCTCAGTGGAACGAA AAC-3′. The purified PCR product was electroporated into MG1655 cells harboring the pKD46 plasmid, and the deletion mutant was selected on LB plates with kanamycin at 37°C.

### Determination of Minimal Inhibitory Concentration (MIC) Values

The MIC values were examined on agar plates with the agar dilution method according to the Clinical Laboratory Standards Institute guidelines (Wikler and CLSI, 2018). The Müller-Hinton agar (Difco, United States) plates were prepared by adding antimicrobial agents in two-fold serial dilutions, resulting in plates containing final concentrations of 512 μg/ml to 7.8 ng/ml. *E. coli* cells were grown in the Mueller-Hinton broth to a McFarland turbidity standard of 0.5 (approximately 1.5 × 10^8^ cells/ml). Cultures were diluted 10-fold with Mueller-Hinton broth to reach a final concentration of 10^7^ cells/ml. A 10 μl volume was spotted on the plates. After incubation at 37°C for 20 h, the MIC value was determined. The MIC is defined as the lowest concentration of an antibiotic preventing the lawn growth of cells.

### Measurement of Bacterial Growth

Stationary phase cells grown in LB medium were serially diluted from 10^8^ to 10^4^ cells/ml in 10-fold dilutions. Aliquots of 2 μl were spotted onto LB plates supplemented with 2% SDS, 6% ethanol, 750 mM NaCl, 64 μg/ml vancomycin, or 20 μg/ml chlorophenyl red-β-d-galactopyranoside (CPRG). To determine the effect of the C-terminal domain of OmpA on antibiotic sensitivity, cells were spotted onto LB plates containing 6 μg/ml ampicillin, 2.5 μg/ml cefalotin, 6 μg/ml choramphenicol, 150 μg/ml clindamycin, or 5 μg/ml rifampicin. After 8–20 h incubation at 37°C, photographs of the plates were taken with a digital camera EOS 100D (Canon Inc., Tokyo, Japan). Isopropyl-β-d-thiogalactopyranoside of 50 μM was added when experiments using CPRG were performed.

### Determination of Membrane Integrity Using Fluorescent Chemicals

For fluorescence imaging, stationary phase cells of wild-type and *ompA ompC ompF* triple mutants were inoculated into LB medium. When the A_600_ reached 2, cells were stained with FM4-64 [N-(3-triethylammoniumpropyl)-4-(*p*-diethylaminophenylhexatrienyl)-pyridinium dibromide], propidium iodide (red), DAPI (4′,6-diamidino-2-phenylindole) (blue), and SYTOX green (green), and then spotted on a 1% agarose pad prepared in phosphate buffered saline (PBS). Cells were visualized using a Nikon Eclipse Ni microscope (Nikon, Japan).

## Results

### The Effect of Porins on Antibiotic Resistance

**FIGURE 1:**
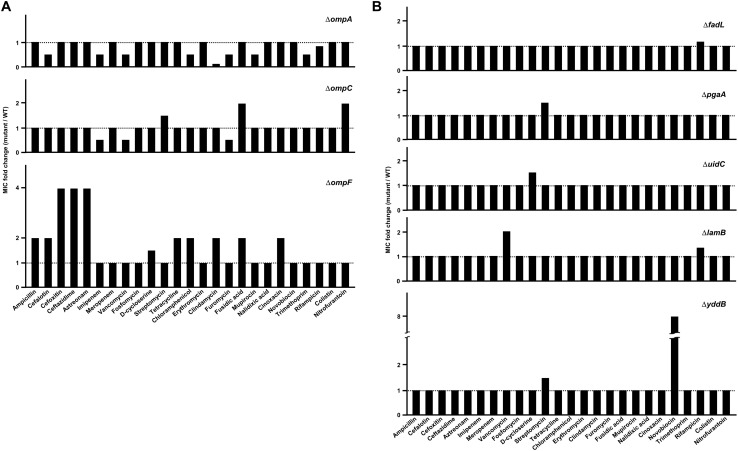
A systematic examination of the effects of porins on antibiotic resistance. **(A)** The MICs of various antibiotics were measured in the wild-type or mutant strain defective for OmpA, OmpC, or OmpF. The relative MIC value in the mutant cells compared to the wild-type cells is shown. **(B)** The MICs of various antibiotics were measured in the wild-type or mutant strain defective for the indicated specific porin. The relative MIC value in the mutant cells compared to the wild-type cells is shown.

To perform a systematic analysis of the effects of porins on antibiotic resistance in *E. coli*, we constructed the deletion mutants for all porin genes of *E. coli* and measured the corresponding MIC values of various antibiotics from different classes (Supplementary Table S3). The *ompA* mutant exhibited an increased susceptibility to many antibiotic classes, including β-lactams, glycopeptides, amphenicols, and licosamides (Figure 1A). There was no antibiotic with an increased MIC for the *ompA* mutant compared to that of wild-type cells. On the other hand, the *ompF* mutant was resistant to many antibiotics belonging to various classes, including β-lactams, amphenicols, tetracyclines, licosamides, steroides, and quinolones (Figure 1A). The *ompF* mutant did not decrease the MIC value of any antibiotic compared to that for the wild-type background. These results suggest that many antibiotics could be transported into the periplasm via OmpF. Notably, the *ompC* mutant was sensitive to some antibiotics, such as imipenem, vancomycin, and furomycin, but it was resistant to other antibiotics, such as streptomycin, fusidic acid, and nitrofurantoin (Figure 1A), suggesting that OmpC is involved in the transport of antibiotics, but has various sensitivity to different classes of these compounds. Therefore, these results indicate that non-specific porins (OmpF, OmpA, and OmpC) play a distinct role in antibiotic resistance.

**FIGURE 2:**
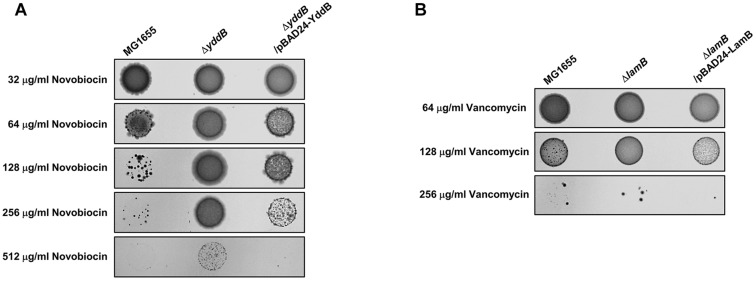
Complemetation of the antibiotic-resistant phenotype of the *yddB* or *lamB* mutant. **(A)** The MICs of novobiocin were examined in 0.01% arabinose-containing agar plates with the agar dilution method according to the Clinical Laboratory Standards Institute guidelines in the wild-type or *yddB* mutant with or without a pBAD-YbbB plasmid. After incubation at 37°C for 20 h, photographs of the plates were taken with a digital camera. **(B)** The MICs of vancomycin were examined in 0.01% arabinose-containing agar plates with the agar dilution method in the wild-type or *lamB* mutant with or without a pBAD-LamB plasmid.

Most specific porins did not affect the MICs of the tested antibiotics (Supplementary Table S1), but several specific porins are associated with the passive transport of some antibiotics (Figure 1B). The *fadL*, *pgaA*, *uidC*, or *lamB* mutant was resistant to rifampicin, streptomycin, d-cycloserine, or vancomycin, respectively. In particular, the *yddB* mutant showed an eight-fold increase in the MIC of novobiocin (Figure 1B). The ectopic expression of the *yddB* gene using the pBAD plasmid with an arabinose-inducible promoter restored the MIC value to the wild-type level (Figure 2A). Similarly, the MIC of vancomycin for the *lamB* mutant was restored to the wild-type level by ectopic expression of the *lamB* gene in the *lamB* mutant (Figure 2B), suggesting that YddB and LamB are the specific porins that are involved in the passive transport of novobiocin and vancomycin, respectively. Therefore, our systematic analyses of the mutants of all specific porins of *E. coli* revealed that most specific porins are not involved in antibiotic resistance. However, several specific porins were shown to be related to the outer membrane penetration of several antibiotics. Specifically, the novel specific porin YddB, playing a major role in the passive transport of novobiocin, was identified.

### The Effect of Porins on the Envelope Stress Response

**FIGURE 3:**
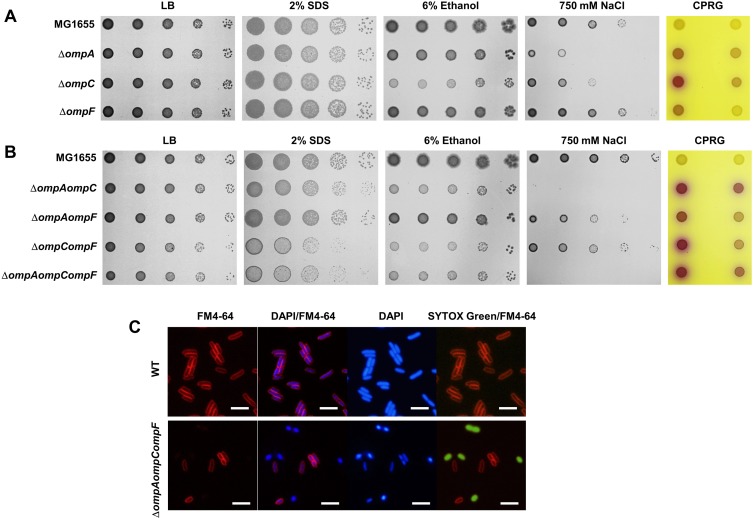
The effect of non-specific porins on envelope stress responses and membrane integrity. **(A)** The wild-type, *ompA*, *ompC*, or *ompF* mutant cells were serially diluted from 10^8^ to 10^4^ cells/ml in 10-fold steps and spotted onto a LB plate or LB plates with the addition of 2% SDS, 6% ethanol, 750 mM NaCl, or 20 μg/ml CPRG as indicated. **(B)** The wild-type, *ompA ompC*, *ompA ompF*, *ompC ompF*, or *ompA ompC ompF* mutant cells were serially diluted 10-fold from 10^8^ to 10^4^ cells/ml and spotted onto a LB plate or LB plates with the addition of 2% SDS, 6% ethanol, 750 mM NaCl, or 20 μg/ml CPRG as indicated. **(C)** The wild-type or *ompA ompC ompF* triple mutant cells grown in LB medium were stained with FM4-64 (red), DAPI (blue), and SYTOX green (green), and then spotted on a 1% agarose pad. Cells were visualized using a Nikon Eclipse Ni microscope. Bars, 0.5 μm.

To elucidate the mechanism of the *ompA* mutant conferring sensitivity to various antibiotics, we examined the effect of porins on envelope stress responses. Although the specific porin mutants were not influenced by envelope stresses, including SDS, ethanol, and salt stresses (Supplementary Figure S1), the non-specific porins, especially OmpA and OmpC, were strongly associated with the envelope stress response (Figure 3). The *ompA* mutant showed a significantly enhanced sensitivity to salt stress. Furthermore, the *ompC* mutant showed slightly increased sensitivity to salt stress. Under ethanol stress, only the *ompC* mutant showed a strong retardation of cell growth (Figure 3A). Three mutant strains did not show any significant difference in growth compared to the wild type strain under SDS stress. These results indicate that OmpA and OmpC play an important role in the envelope stress response.

To analyze the roles of non-specific porins in the envelope stress response in more detail, we constructed double and triple mutants of non-specific porins. Under salt stress, the *ompA* ompC double mutant showed a strong growth defect similar to that of the *ompA ompC ompF* triple mutant (Figure 3B). Additionally, the growth inhibition of the *ompA* or *ompC* mutant under salt stress was not enhanced by an additional deletion of the *ompF* gene. These results imply that OmpA and OmpC are of significant importance for the salt stress response. In ethanol stress, the ethanol-sensitive phenotype of the *ompC* mutant was not increased by the additional deletion of the *ompA* or *ompF* gene (Figure 3B), suggesting that only OmpC is associated with the ethanol stress response. Under SDS stress, the *ompC ompF* double mutant showed an enhanced sensitivity to this condition, although the single deletion mutants of non-specific porins were not affected by this stress. The growth defect was not increased by the additional deletion of the *ompA* gene (Figure 3B). Therefore, OmpC and OmpF seem to be associated with the SDS stress response. However, because the *ompA ompC* double mutant was slightly more sensitive to SDS stress than the *ompC* mutant, OmpA also seems to play a minor effect. In summary, these results show that all of the non-specific porins are involved in the envelope stress response, but their roles are different. OmpC seems to play an important role in all stress responses tested, whereas OmpA and OmpF seem to be mainly associated with salt stress and SDS stress responses, respectively. Consistent with the results on antibiotic resistance, these results show distinct roles between non-specific porins.

### The Effect of Porins on Membrane Permeability

The importance of non-specific porins on the envelope stress response imply that non-specific porins may affect the membrane permeability. To test this assumption, we performed a LacZ assay using CPRG, a substrate of LacZ that cannot penetrate the *E. coli* envelope (Paradis-Bleau et al., 2014; Choi et al., 2017). CPRG is able to penetrate the membrane of the cells with increased membrane permeability, where cytoplasmic LacZ degrades it to chlorophenyl red. These cells form red colonies by chlorophenyl red. Expectedly, the membrane permeability test using CPRG showed that only three mutants defective for non-specific porins exhibited increased membrane permeability (Figure 3 and Supplementary Figure S2). The *ompC* mutant that is involved in all envelope stress responses tested, showed the strongest red phenotype. We also experimentally examined the membrane permeability through experiments using the cell-impermeable, fluorescent DNA dye SYTOX green (Roth et al., 1997). The wild-type cells were not stained with the green fluorescent SYTOX green dye, whereas the *ompA ompC ompF* triple mutant showed a large increase in the intracellular SYTOX green signal (Figure 3C), indicating an increase of membrane permeability. Interestingly, the SYTOX green-stained cells of the *ompA ompC ompF* triple mutant were not stained with the amphiphilic membrane dye FM4-64. This result implies that the big change at the OM happens in the triple mutant. We examined the presence of dead cells for this mutant strain, because the *ompA ompC ompF* triple mutant exhibited a growth defect even in LB medium (Figure 3B). Live–dead staining using the cell viability stain propidium iodide (Meeske et al., 2016), showed decreased cell viability in the *ompA ompC ompF* triple mutant cells compared to the wild-type cells (Supplementary Figure S3). Therefore, these results indicate that non-specific porins affect the membrane permeability and bacterial viability.

### The Importance of the C-Terminal Domain of OmpA in Its Function

**FIGURE 4:**
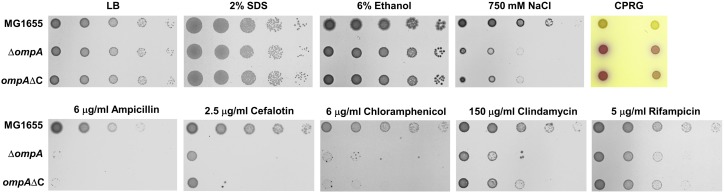
The importance of the C-terminal region of OmpA on its function. The wild-type, *ompA*, or *ompA*ΔC mutant cells were serially diluted from 10^8^ to 10^4^ cells/ml in 10-fold steps and spotted onto a LB plate or LB plates with the addition of 2% SDS, 6% ethanol, 750 mM NaCl, 20 μg/ml CPRG, 6 μg/ml ampicillin, 2.5 μg/ml cefalotin, 6 μg/ml choramphenicol, 150 μg/ml clindamycin, or 5 μg/ml rifampicin as indicated.

OmpA has a flexible C-terminal domain that non-covalently interacts with peptidoglycan (Samsudin et al., 2016). To determine whether this domain is important for the effect of OmpA on antibiotic resistance and the envelope stress response, we constructed the mutant strain (*ompA*ΔC) that is chromosomally deleted for the C-terminal domain of OmpA. Notably, all of the phenotypes of the *ompA* mutant were phenocopied by the *ompA*ΔC mutant (Figure 4). For example, the *ompA*ΔC mutant was sensitive to salt stress to an extent similar to the *ompA* mutant and showed a phenotype almost identical to the *ompA* mutant in the antibiotic susceptibility test. These results suggest that the effect of OmpA on antibiotic resistance and the envelope stress response is entirely dependent on the C-terminal domain of OmpA. The loss of the interaction with peptidoglycan might weaken the turgor of the bacterial envelope, which can induce the increased membrane permeability and antibiotic penetration.

### The Effect of Double and Triple Mutants of Non-specific Porins on Antibiotic Resistance

**FIGURE 5:**
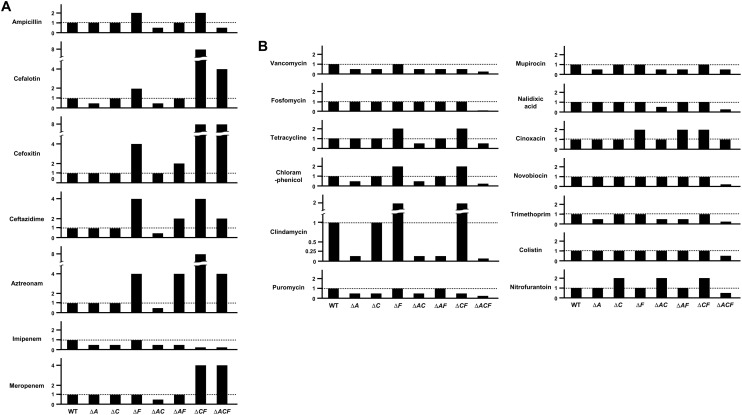
The effects of single-, double-, and triple-mutants of non-specific porins on antibiotic resistance. **(A)** The MICs of β-lactam antibiotics were measured in the wild-type strain or single-, double-, and triple-mutants of OmpA, OmpC, and OmpF. The relative MIC value in the mutant cells compared to the wild-type cells was shown. **(B)** The MICs of non-β-lactam antibiotics were measured in the wild-type strain or single-, double-, and triple-mutants of OmpA, OmpC, and OmpF. The relative MIC value in the mutant cells compared to the wild-type cells was shown: WT, MG1655; ΔA, Δ*ompA*; ΔC, Δ*ompC*; ΔF, Δ*ompF*, ΔAC, Δ*ompA ompC*; ΔAF, Δ*ompA ompF*; ΔCF, Δ*ompC ompF*; ΔACF, Δ*ompA ompC ompF*.

Our results showed that specific porins are only associated with the passive transport of antibiotics, whereas non-specific porins are involved in membrane permeability as well as antibiotic transport, indicating the functional diversity of non-specific porins. To understand the various functions of non-specific porins in more detail, we analyzed the antibiotic susceptibility of double and triple mutants of the *ompA*, *ompC*, and *ompF* genes (Figure 5). Notably, phenotype patterns of these mutants could be divided into two groups: β-lactams and other antibiotics. Besides ampicillin and imipenem, the susceptibilities to most β-lactam antibiotics were strongly affected by OmpF. The MIC of most β-lactams was the lowest in the *ompA ompC* double mutant and the additional deletion of the *ompF* gene strongly increased the MIC in the *ompA ompC* double mutant; consequently, the MICs in the *ompA ompC ompF* triple mutant were 2- to 8-fold higher than those in the wild-type strain (Figure 5A). OmpC also seems to be important for the transport of β-lactam antibiotics. The additional deletion of the *ompC* gene increased the MICs in the *ompF* mutant up to four times. Therefore, these results suggest that the transport of β-lactam antibiotics by OmpF and OmpC is the most important factor in the bacterial susceptibility to most β-lactam antibiotics. Ampicillin and imipenem are exceptional cases. Although OmpF is a porin that plays an important role in the transport of ampicillin (Delcour, 2009), the MIC in the *ompA ompC ompF* triple mutant was 2-fold lower than that in the wild-type strain (Figure 5A). The MIC of imipenem was also 4-fold lower in the triple mutant than in the wild-type strain. These results imply that the effect of non-specific porins on membrane permeability may be a more important factor in the bacterial susceptibility to ampicillin and imipenem.

In all non-β-lactam antibiotics tested, the strain with the lowest MIC value was the *ompA ompC ompF* triple mutant (Figure 5B). Even in the case of antibiotics exhibiting higher MICs in the *ompF ompC* double mutant than in the wild-type strain, the additional deletion of the *ompA* gene decreased the MICs up to 32 times. The example of clindamycin demonstrates this pattern clearly. Although the MIC of clindamycin was 2-fold higher in the *ompF* or *ompF ompC* double mutant than in the wild-type strain, the MICs in the *ompF ompA* double or *ompF ompC ompA* triple mutant was 16- or 32-fold lower than those of the wild-type strain (Figure 5B). Therefore, these results suggest that in non-β-lactam antibiotics, the effect of non-specific porins, particularly OmpA, on membrane integrity is more important for antibiotic susceptibility than their roles in antibiotic transport. In conclusion, analyses of the effects of double and triple mutants on antibiotic resistance revealed that two roles of non-specific porins, antibiotic transport and the maintenance of membrane integrity, differently affect the antibiotic susceptibility depending on the kind of antibiotic. The transport of antibiotics via non-specific porins, particularly OmpF and OmpC, strongly affects the susceptibility to most β-lactam antibiotics, whereas the maintenance of the membrane integrity by non-specific porins seems to be more important for the susceptibility to non-β-lactam antibiotics.

## Discussion

In this study, we performed a systematic analysis examining the roles of all porins of *E. coli* in antibiotic susceptibility. Despite the importance of porins for antibiotic resistance, a systematic study about porins has not been attempted previously. Our study shows the complexity of porin-mediated antibiotic resistance. OmpA plays an important role in the maintenance of membrane integrity, rather than the transport of antibiotics (Figure 1). The C-terminal domain is essential for these OmpA functions (Figure 4). Although OmpF is associated with both antibiotic transport and membrane permeability, the *ompF* mutant exhibits antibiotic resistance phenotypes (Figure 1A). Increased susceptibility to antibiotic was not found. These results may be due to a minor effect of OmpF on membrane integrity (Figure 3). OmpF is known to be a major porin for the OM permeability for β-lactams (Delcour, 2009), which is also supported by our results. The *ompF* mutant showed significantly increased resistance to several β-lactam antibiotics (Figure 1A). Additionally, in many β-lactam antibiotics, the *ompA ompC* mutant had the lowest MICs. However, the MICs were dramatically increased with the additional deletion of the *ompF* gene (Figure 5). These results show the importance of OmpF in the passive transport of β-lactam antibiotics. OmpC seems to play an important role in both antibiotic transport and membrane permeability (Figures 1, 4, 5). Deletion of the *ompC* gene increased the MICs of some antibiotics, but it decreased the MICs of other antibiotics (Figure 1). OmpC seems to be associated with the transport of many β-lactam antibiotics (Figure 5), which is consistent with previous results (Nikaido et al., 1983; Lou et al., 2011; Kojima and Nikaido, 2014). Furthermore, OmpC plays an important role in the adaptation to various envelope stresses (Figure 3). Therefore, these results demonstrate the distinct roles of three non-specific porins. A previous study showed that the expression level of OmpF was lower than those of OmpA and OmpC (Sato et al., 2000). Our results show that OmpA and OmpC plays an important role in the maintenance of membrane integrity, whereas OmpF is not strongly involved in the maintenance of membrane integrity. Therefore, there is a possibility that *E. coil* differentially expresses three non-specific porins based on their distinct roles in the maintenance of membrane integrity. All specific porins do not seem to be involved in the envelope stress response (Supplementary Figure S1). Therefore, there is no case with increased antibiotic susceptibility in the mutant defective for specific porin (Figure 1B and Supplementary Table S3). Several specific porins seem to be involved in the passive transport of several antibiotics (Figure 1B). Therefore, *E. coli* porins can be classified into three groups according to their roles in antibiotic transport and membrane integrity: antibiotic transport-related specific porins (LamB, YddB, etc.), membrane integrity-related non-specific porin (OmpA), and porins associated with both antibiotic transport and membrane integrity (OmpC and OmpF). The functional diversity of porins might indicate why the effects of porins on antibiotic resistance differ so widely depending on the kind of porin. The efflux systems strongly affect antibiotic resistance (Li et al., 2015). Therefore, further studies are required to analyze of the roles of porins in the absence of efflux systems and examine the effects of porins on expression of the efflux systems.

Through our systematic study on *E. coli* specific porins, we identified a novel porin YddB which seems to be involved in the passive transport of novobiocin across the OM. YddB is an outer membrane protein that is predicted to have a β-barrel structure, but its function is unknown. The *yddB* mutant displays a dramatically decreased susceptibility to novobiocin compared to the wild type strain (Figure 1). The MIC of novobiocin was not altered by the deletion of any porin gene (Supplementary Table S3); therefore, YddB seems to be a major porin that is responsible for the OM penetration of novobiocin. Besides particular examples, such as YddB and LamB, the role of specific porins for antibiotic transport is of minor importance. Most β-lactam antibiotics are transported with non-specific porins, especially OmpF and OmpC, and most non-β-lactam antibiotics seem to penetrate the OM in a porin-independent manner. However, because a synergistic effect among specific porins cannot be excluded, further experiments are required to analyze whether antibiotic resistance is affected by double or triple deletions of specific porins.

In this study, we find that all non-specific porins, OmpA, OmpC, and OmpF, are involved in the maintenance of membrane integrity, despite a large diversity of their roles and influences. OmpA is known to affect membrane integrity through non-covalent interaction of its C-terminal periplasmic domain with peptidoglycan (Samsudin et al., 2016). The role of the C-terminal domain seems to be critical for the role of OmpA in antibiotic resistance as well as the maintenance of membrane integrity (Figure 4). OmpC is known as osmoporin and the expression of *ompC* increases under high osmolarity conditions (Pratt et al., 1996). The *ompC* mutant was sensitive to various envelope stresses (Kaeriyama et al., 2006; Wang et al., 2007; Figure 3), but the exact reason for this phenotype is unknown. A recent study showed that OmpC forms a complex with MlaA and may function in the removal of phospholipids from the OM (Chong et al., 2015). MlaA is a component of the Mla pathway mediating the retrograde transport of phospholipids mislocalized in the outer leaflet of the OM for the maintenance of OM lipid asymmetry (Choi and Lee, 2019). Because the mislocalization of phospholipids in the outer leaflet of the OM weakens the OM integrity (Sutterlin et al., 2016), the role of OmpC in the retrograde transport of phospholipids can be one of the reasons for the diverse OmpC-associated phenotypes. Our study showed that OmpF is also associated with membrane integrity regulation (Figure 3). Because there is no report showing the relationship between OmpF and membrane integrity, the mechanism of this effect remains to be elucidated.

## Conclusion

In conclusion, our systematic study shows two possible roles of porins, the transport of antibiotics and the membrane integrity regulation, which differently affect antibiotic resistance, and the distinct role of each porin. This provides an important insight to understand not only the role of each porin as a functionally important component of the outer membrane, but also its specific role in the survival of the pathogen under the action of antibiotics.

## Data Availability

All datasets generated for this study are included in the manuscript and/or the Supplementary Files.

## Author Contributions

C-RL contributed to the conception and design of the experiments. C-RL and UC researched and wrote the manuscript.

## Conflict of Interest Statement

The authors declare that the research was conducted in the absence of any commercial or financial relationships that could be construed as a potential conflict of interest.

## References

[B1] Bornet C. Davin-Regli A. Bosi C. Pages J. M. Bollet C. (2000). Imipenem resistance of *Enterobacter aerogenes* mediated by outer membrane permeability. *J. Clin. Microbiol.* 38 1048–1052. 10698994 10.1128/jcm.38.3.1048-1052.2000 PMC86335

[B2] Choi U. Lee C. R. (2019). Antimicrobial agents that inhibit the outer membrane assembly machines of gram negative bacteria. *J. Microbiol. Biotechnol.* 29 1–10. 10.4014/jmb.1804.03051 29996592

[B3] Choi U. Park Y. H. Kim Y. R. Seok Y. J. Lee C. R. (2016). Increased expression of genes involved in uptake and degradation of murein tripeptide under nitrogen starvation in *Escherichia coli*. *FEMS Microbiol. Lett.* 363:fnw136. 10.1093/femsle/fnw136 27231238

[B4] Choi U. Park Y. H. Kim Y. R. Seok Y. J. Lee C. R. (2017). Effect of the RNA pyrophosphohydrolase RppH on envelope integrity in *Escherichia coli*. *FEMS Microbiol. Lett.* 364:fnx152. 10.1093/femsle/fnx152 28859318

[B5] Chong Z. S. Woo W. F. Chng S. S. (2015). Osmoporin OmpC forms a complex with MlaA to maintain outer membrane lipid asymmetry in *Escherichia coli*. *Mol. Microbiol.* 98 1133–1146. 10.1111/mmi.13202 26314242

[B6] Datsenko K. A. Wanner B. L. (2000). One-step inactivation of chromosomal genes in *Escherichia coli* K-12 using PCR products. *Proc. Natl. Acad. Sci. U.S.A.* 97 6640–6645. 10.1073/pnas.120163297 10829079 PMC18686

[B7] Delcour A. H. (2009). Outer membrane permeability and antibiotic resistance. *Biochim. Biophys. Acta* 1794 808–816. 10.1016/j.bbapap.2008.11.005 19100346 PMC2696358

[B8] Henderson J. C. Zimmerman S. M. Crofts A. A. Boll J. M. Kuhns L. G. Herrera C. M. (2016). The power of asymmetry: architecture and assembly of the Gram-negative outer membrane lipid bilayer. *Annu. Rev. Microbiol.* 70 255–278. 10.1146/annurev-micro-102215-095308 27359214 PMC12914872

[B9] Iyer R. Moussa S. H. Durand-Reville T. F. Tommasi R. Miller A. (2018). *Acinetobacter baumannii* OmpA is a selective antibiotic permeant porin. *ACS Infect. Dis.* 4 373–381. 10.1021/acsinfecdis.7b00168 29260856

[B10] Kaeriyama M. Machida K. Kitakaze A. Wang H. Lao Q. Fukamachi T. (2006). OmpC and OmpF are required for growth under hyperosmotic stress above pH 8 in *Escherichia coli*. *Lett. Appl. Microbiol.* 42 195–201. 10.1111/j.1472-765X.2006.01845.x 16478504

[B11] Koebnik R. Locher K. P. Van Gelder P. (2000). Structure and function of bacterial outer membrane proteins: barrels in a nutshell. *Mol. Microbiol.* 37 239–253. 10.1046/j.1365-2958.2000.01983.x 10931321

[B12] Kojima S. Nikaido H. (2014). High salt concentrations increase permeability through OmpC channels of *Escherichia coli*. *J. Biol. Chem.* 289 26464–26473. 10.1074/jbc.M114.585869 25086034 PMC4176201

[B13] Lee C. R. Cho I. H. Jeong B. C. Lee S. H. (2013). Strategies to minimize antibiotic resistance. *Int. J. Environ. Res. Public Health* 10 4274–4305. 10.3390/ijerph10094274 24036486 PMC3799537

[B14] Lee J. Park Y. H. Kim Y. R. Seok Y. J. Lee C. R. (2015). Dephosphorylated NPr is involved in an envelope stress response of *Escherichia coli*. *Microbiology* 161 1113–1123. 10.1099/mic.0.000056 25701731 PMC4635465

[B15] Li X. Z. Plesiat P. Nikaido H. (2015). The challenge of efflux-mediated antibiotic resistance in Gram-negative bacteria. *Clin. Microbiol. Rev.* 28 337–418. 10.1128/CMR.00117-14 25788514 PMC4402952

[B16] Lou H. Chen M. Black S. S. Bushell S. R. Ceccarelli M. Mach T. (2011). Altered antibiotic transport in OmpC mutants isolated from a series of clinical strains of multi-drug resistant *E. coli*. *PLoS One* 6:e25825. 10.1371/journal.pone.0025825 22053181 PMC3203869

[B17] Mach T. Neves P. Spiga E. Weingart H. Winterhalter M. Ruggerone P. (2008). Facilitated permeation of antibiotics across membrane channels-interaction of the quinolone moxifloxacin with the OmpF channel. *J. Am. Chem. Soc.* 130 13301–13309. 10.1021/ja803188c 18788798

[B18] Meeske A. J. Riley E. P. Robins W. P. Uehara T. Mekalanos J. J. Kahne D. (2016). SEDS proteins are a widespread family of bacterial cell wall polymerases. *Nature* 537 634–638. 10.1038/nature19331 27525505 PMC5161649

[B19] Moya-Torres A. Mulvey M. R. Kumar A. Oresnik I. J. Brassinga A. K. (2014). The lack of OmpF, but not OmpC, contributes to increased antibiotic resistance in *Serratia marcescens*. *Microbiology* 160 1882–1892. 10.1099/mic.0.081166-0 25015362

[B20] Nikaido H. (2003). Molecular basis of bacterial outer membrane permeability revisited. *Microbiol. Mol. Biol. Rev*. 67 593–656. 10.1128/MMBR.67.4.593-656.2003 14665678 PMC309051

[B21] Nikaido H. Rosenberg E. Y. Foulds J. (1983). Porin channels in *Escherichia coli*: studies with β-lactams in intact cells. *J. Bacteriol.* 153 232–240. 6294048 10.1128/jb.153.1.232-240.1983 PMC217361

[B22] Okamoto K. Gotoh N. Nishino T. (2001). *Pseudomonas aeruginosa* reveals high intrinsic resistance to penem antibiotics: penem resistance mechanisms and their interplay. *Antimicrob. Agents Chemother.* 45 1964–1971. 10.1128/AAC.45.7.1964-1971.2001 11408209 PMC90586

[B23] O’Shea R. Moser H. E. (2008). Physicochemical properties of antibacterial compounds: implications for drug discovery. *J. Med. Chem*. 51 2871–2878. 10.1021/jm700967e 18260614

[B24] Pages J. M. James C. E. Winterhalter M. (2008). The porin and the permeating antibiotic: a selective diffusion barrier in Gram-negative bacteria. *Nat. Rev. Microbiol.* 6 893–903. 10.1038/nrmicro1994 18997824

[B25] Paradis-Bleau C. Kritikos G. Orlova K. Typas A. Bernhardt T. G. (2014). A genome-wide screen for bacterial envelope biogenesis mutants identifies a novel factor involved in cell wall precursor metabolism. *PLoS Genet.* 10:e1004056. 10.1371/journal.pgen.1004056 24391520 PMC3879167

[B26] Pratt L. A. Hsing W. Gibson K. E. Silhavy T. J. (1996). From acids to osmZ: multiple factors influence synthesis of the OmpF and OmpC porins in *Escherichia coli*. *Mol. Microbiol.* 20 911–917. 10.1111/j.1365-2958.1996.tb02532.x 8809744

[B27] Roth B. L. Poot M. Yue S. T. Millard P. J. (1997). Bacterial viability and antibiotic susceptibility testing with SYTOX green nucleic acid stain. *Appl. Environ. Microbiol.* 63 2421–2431. 9172364 10.1128/aem.63.6.2421-2431.1997 PMC168536

[B28] Samsudin F. Ortiz-Suarez M. L. Piggot T. J. Bond P. J. Khalid S. (2016). OmpA: a flexible clamp for bacterial cell wall attachment. *Structure* 24 2227–2235. 10.1016/j.str.2016.10.009 27866852

[B29] Sato M. Machida K. Arikado E. Saito H. Kakegawa T. Kobayashi H. (2000). Expression of outer membrane proteins in *Escherichia coli* growing at acid pH. *Appl. Environ. Microbiol.* 66 943–947. 10.1128/AEM.66.3.943-947.2000 10698756 PMC91927

[B30] Schulz G. E. (2002). The structure of bacterial outer membrane proteins. *Biochim. Biophys. Acta* 1565 308–317. 10.1016/S0005-2736(02)00577-1 12409203

[B31] Smani Y. Fabrega A. Roca I. Sanchez-Encinales V. Vila J. Pachon J. (2014). Role of OmpA in the multidrug resistance phenotype of *Acinetobacter baumannii*. *Antimicrob. Agents Chemother.* 58 1806–1808. 10.1128/AAC.02101-13 24379205 PMC3957889

[B32] Sugawara E. Kojima S. Nikaido H. (2016). *Klebsiella pneumoniae* major porins OmpK35 and OmpK36 allow more efficient diffusion of β-lactams than their *Escherichia coli* homologs OmpF and OmpC. *J. Bacteriol.* 198 3200–3208. 10.1128/JB.00590-16 27645385 PMC5105900

[B33] Sugawara E. Nikaido H. (1992). Pore-forming activity of OmpA protein of *Escherichia coli*. *J. Biol. Chem.* 267 2507–2511. 1370823

[B34] Sutterlin H. A. Shi H. May K. L. Miguel A. Khare S. Huang K. C. (2016). Disruption of lipid homeostasis in the Gram-negative cell envelope activates a novel cell death pathway. *Proc. Natl. Acad. Sci. U.S.A.* 113 E1565–E1574. 10.1073/pnas.1601375113 26929379 PMC4801249

[B35] Wang Y. Wang L. Sun Y. Chen Y. Zhu L. Guo L. (2007). Disrupted OmpC causes osmosis sensitivity of *Escherichia coli* in alkaline medium. *J. Genet. Genomics* 34 1131–1138. 10.1016/S1673-8527(07)60129-5 18155626

[B36] Wikler M. A. CLSI (2018). *Methods for Dilution Antimicrobial Susceptibility Tests for Bacteria That Grow Aerobically; Approved Standard*, 11th Edn Wayne, PA: Clinical and Laboratory Standards Institute.

[B37] Ziervogel B. K. Roux B. (2013). The binding of antibiotics in OmpF porin. *Structure* 21 76–87. 10.1016/j.str.2012.10.014 23201272 PMC3545085

