# Supplementary material for: Distinct Roles of Outer Membrane Porins in Antibiotic Resistance and Membrane Integrity in Escherichia coli
Source: Front Microbiol. 2019 Apr 30;10:953. doi: 10.3389/fmicb.2019.00953 (PMC6503746; doi:10.3389/fmicb.2019.00953)
Supplement: Supplementary file 1 [file Data_Sheet_1.docx]

Supplementary Material

# Supplementary Figures and Tables

## Supplementary Figures


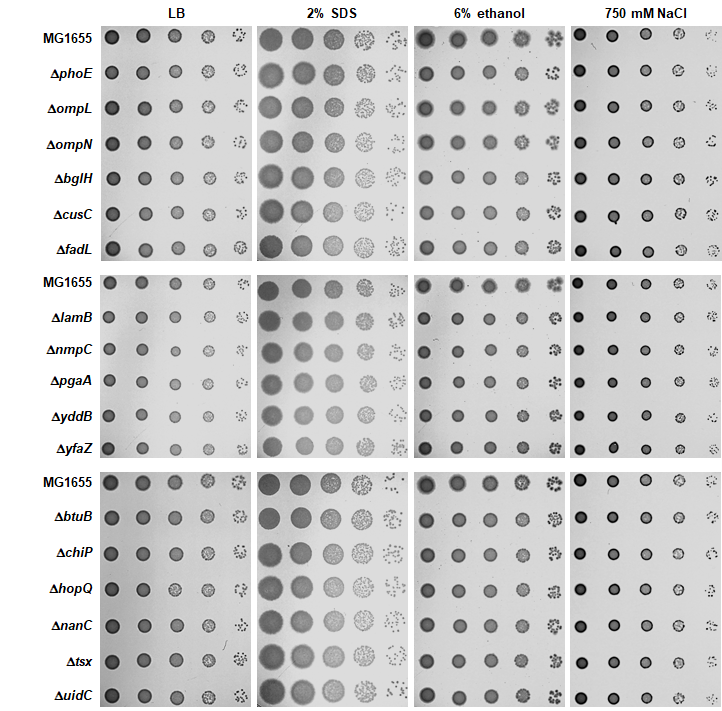


**Supplementary Figure 1.** The effect of specific porins on envelope stress responses. The indicated cells were serially 10-fold diluted from 10^8^ to 10^4^ cells ml^−1^ and spotted onto a LB plate or LB plates with the addition of 2% SDS, 6% ethanol, or 750 mM NaCl as indicated.


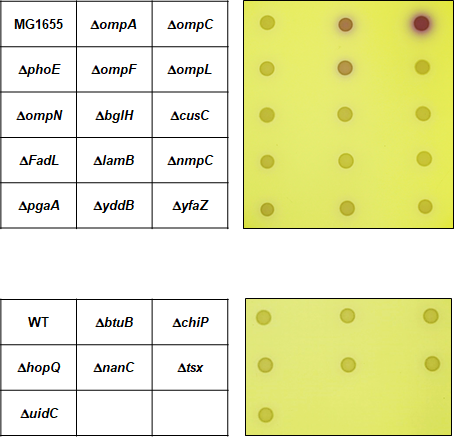


**Supplementary Figure 2.** The effect of porins on membrane integrity. The indicated cells were serially 10-fold diluted from 10^8^ to 10^4^ cells ml^−1^ and spotted onto a LB plate or LB plates with the addition of 20 μg/ml CPRG as indicated.


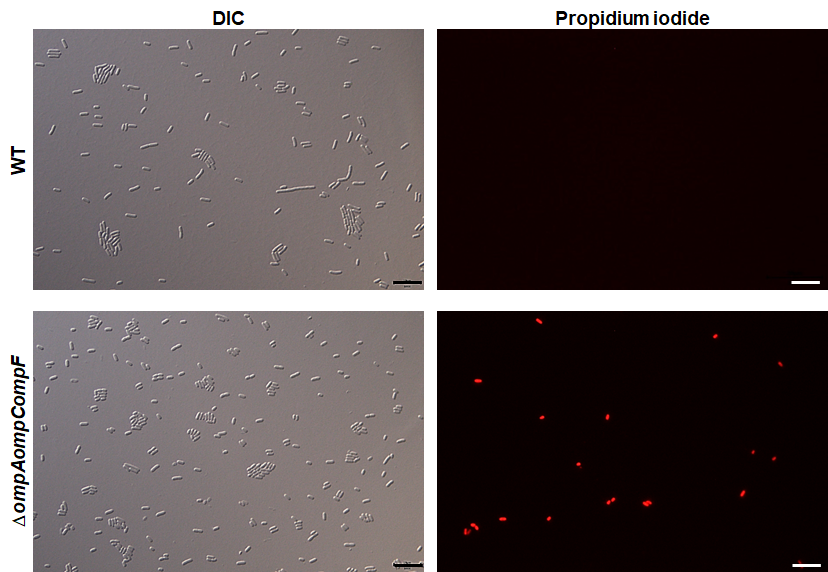


**Supplementary Figure 3.** Live–dead staining using propidium iodide. The wild-type or *ompA ompC ompF* triple mutant cells grown in LB medium were stained with propidium iodide (red), and then spotted on a 1% agarose pad made with PBS. Cells were visualized using a Nikon Eclipse Ni microscope. Bars, 10 μm.

## Supplementary Tables

**Table S1.** ***Escherichia coli* strains and plasmids used in this study.**

| Strain or plasmid | Genotype or phenotype | Source or Reference |
| --- | --- | --- |
| **Strains** |  |  |
| MG1655 | F^-^ λ^-^ *ilvG*^-^ *rfb*-50 *rph*-1. Wild type *E. coli* K-12 | (Blattner et al., 1997) |
| MG1655 Δ*ompA* | MG1655 *ompA:: frt* | This study |
| MG1655 Δ*ompC* | MG1655 *ompC:: frt* | This study |
| MG1655 Δ*phoE* | MG1655 *phoE:: frt* | This study |
| MG1655 Δ*ompF* | MG1655 *ompF:: frt* | This study |
| MG1655 Δ*ompL* | MG1655 *ompL:: frt* | This study |
| MG1655 Δ*ompN* | MG1655 *ompN:: frt* | This study |
| MG1655 Δ*bglH* | MG1655 *bglH:: frt* | This study |
| MG1655 Δ*fadL* | MG1655 *fadL:: frt* | This study |
| MG1655 Δ*lamB* | MG1655 *lamB:: frt* | This study |
| MG1655 Δ*nmpC* | MG1655 *nmpC:: frt* | This study |
| MG1655 Δ*pgaA* | MG1655 *pgaA:: frt* | This study |
| MG1655 Δ*yddB* | MG1655 *yddB:: frt* | This study |
| MG1655 Δ*yfaZ* | MG1655 *yfaZ:: frt* | This study |
| MG1655 Δ*butB* | MG1655 *btuB:: frt* | This study |
| MG1655 Δ*chiP* | MG1655 *chiP:: frt* | This study |
| MG1655 Δ*hofQ* | MG1655 *hofQ:: frt* | This study |
| MG1655 Δ*nanC* | MG1655 *nanC:: frt* | This study |
| MG1655 Δ*tsx* | MG1655 *tsx:: frt* | This study |
| MG1655 Δ*uidC* | MG1655 *uidC:: frt* | This study |
| MG1655 Δ*ompAompC* | MG1655 *ompC::frt ompA:: frt* | This study |
| MG1655 Δ*ompAompF* | MG1655 *ompF::frt ompA:: frt* | This study |
| MG1655 Δ*ompCompF* | MG1655 *ompF::frt ompC:: frt* | This study |
| MG1655 Δ*ompAompCompF* | MG1655 *ompF::frt ompC::frt ompA:: frt* | This study |
| MG1655 *ompA*Δ*C* | MG1655 *ompA*Δ*C::*Kan^R^ | This study |
|  |  |  |
| **Plasmids** |  |  |
| pBAD24 | Expression vector under control of arabinose-inducible promoter, Cm^r^ | Addgene |
| pBAD-LamB | pBAD24-based expression vector for LamB, Cm^r^ | This study |
| pBAD-YddB | pBAD24-based expression vector for YddB, Cm^r^ | This study |
| pKD13 | Template plasmid for the amplification of the kanamycin-resistance gene bordered by FRT sites, Km^r^ | (Datsenko and Wanner, 2000) |
| pKD46 | λ Red recombinase expression plasmid Ts replicon, Amp^r^ | (Datsenko and Wanner, 2000) |
| pCP20 | FLP helper plasmid Ts replicon, Amp^r^ Cm^r^ | (Datsenko and Wanner, 2000) |

**Table S2.** **Oligonucleotides used in this study**

| **Name** | **Oligonucleotide sequence (5’–3’)** | **Use** |
| --- | --- | --- |
| OmpA-FRT-F | CTCGTTGGAGATATTCATGGCGTATTTTGGATGATAACGAGGCGCAAAAA GTGTAGGCTGGAGCTGCTTC | Deletion |
| OmpA-FRT-R | AAAGGCAAAAAAAACCCCGCAGCAGCGGGGTTTTTCTACCAGACGAGAAC ATTCCGGGGATCCGTCGACC |  |
| OmpC-FRT-F | TGCAGTGGCATAAAAAAGCAAATAAAGGCATATAACAGAGGGTTAATAAC GTGTAGGCTGGAGCTGCTTC |  |
| OmpC-FRT-R | AAAACAATGAAAAAAGGGCCCGCAGGCCCTTTGTTCGATATCAATCGAGA ATTCCGGGGATCCGTCGACC |  |
| PhoE-FRT-F | CGGGACTCTCCCGCTGAATATTCGCGCGTTAATTAAAATCAGGAATGAAA GTGTAGGCTGGAGCTGCTTC |  |
| PhoE-FRT-R | TTAACGGCGTGAGTGAAATGTGCCGGATGCATCACATCCGGCAATATTCA ATTCCGGGGATCCGTCGACC |  |
| OmpF-FRT-F | ATTGACGGCAGTGGCAGGTGTCATAAAAAAAACCATGAGGGTAATAAATA GTGTAGGCTGGAGCTGCTTC |  |
| OmpF-FRT-R | AAACAGGACCAAAGTCCTGTTTTTTCGGCATTTAACAAAGAGGTGTGCTA ATTCCGGGGATCCGTCGACC |  |
| OmpL-FRT-F | TTCCATCCTGGTGGGTGGCGGCCTCCCTACGTTTAAAAAATGGACTTATT GTGTAGGCTGGAGCTGCTTC |  |
| OmpL-FRT-R | ATCAACCGTAGGCCGGATAAGGCGTTTACGCCGCATCCGGCAGTTTCACA ATTCCGGGGATCCGTCGACC |  |
| OmpN-FRT-F | TCCCCGAAATGGGGATAAAAGGCAAATAAAATAACTAAAGGATTTATTCA GTGTAGGCTGGAGCTGCTTC |  |
| OmpN-FRT-R | AGGAGGAAGAATAAGCAGAAAAAGCCCGCCATAACAGCGGGCAGGAGGAT ATTCCGGGGATCCGTCGACC |  |
| BglH-FRT-F | CAGTCTGGATGCGATGCGTTAATTCTTTCTTTGCACCATAAAGGGATATT GTGTAGGCTGGAGCTGCTTC |  |
| BglH-FRT-R | TAAAGCAGCAATTTTTATATTCATCGTAATATTCCTTTCATTAAGTTCCA ATTCCGGGGATCCGTCGACC |  |
| FadL-FRT-F | TACAGCACGTAACATAGTTTGTATAAAAATAAATCATTGAGGTTATGGTC GTGTAGGCTGGAGCTGCTTC |  |
| FadL-FRT-R | GTGGTACCCAACGCGATGCGGTAAGCATCTTTAAAGCCTTCATGTTTCTG ATTCCGGGGATCCGTCGACC |  |
| LamB-FRT-F | GCAATGACTCAGGAGATAGAATGATGATTACTCTGCGCAAACTTCCTCTG GTGTAGGCTGGAGCTGCTTC |  |
| LamB-FRT-R | TCGCCCATGGAGATCGCACCGTGGTCGAGGATACGCAGCATGTGACCGTT ATTCCGGGGATCCGTCGACC |  |
| NmpC-FRT-F | GTTCAAAAAAAGGGCTCACGATGAAAAAATTAACAGTGGCAATTTCTGCT GTGTAGGCTGGAGCTGCTTC |  |
| NmpC-FRT-R | TTGTTGAAGTAATAGGTTGCACCTACATCAACATATTTGACTAAGTCCTG ATTCCGGGGATCCGTCGACC |  |
| PgaA-FRT-F | CTGTAATTAGATACAGAGAGAGATTTTGGCAATACATGGAGTAATACAGG GTGTAGGCTGGAGCTGCTTC |  |
| PgaA-FRT-R | ACTCACCAGCATCAGGAGATATTTATTTCCATTACGTAACATATTTATCC ATTCCGGGGATCCGTCGACC |  |
| YddB-FRT-F | CGCGGTTTTATAAAAACAATAAAAGCCCTGCTGTACGGAGATATAAATAA GTGTAGGCTGGAGCTGCTTC |  |
| YddB-FRT-R | CTCCATGCGAAAACCGGGCGAATTTACCCGGTTAAGTAAAATCCGAACTA ATTCCGGGGATCCGTCGACC |  |
| YfaZ-FRT-F | AATCCCGCAGCTTTGGGAATTTTGATTAATTGCTTGTGGGAAAGGGGATA GTGTAGGCTGGAGCTGCTTC |  |
| YfaZ-FRT-R | AGGCGTAGTAAGAAACTGAATGGCGCGACAGAATGCCGCGCCGGAAGGGA ATTCCGGGGATCCGTCGACC |  |
| BtuB-FRT-F | TAATATTGATGAAACCTGCGGCATCCTTCTTCTATTGTGGATGCTTTACA GTGTAGGCTGGAGCTGCTTC |  |
| BtuB-FRT-R | CTCAACATAACCTGTACCCGGCGTCGTAGTCTGTTTCTGCCAGTCGACAC ATTCCGGGGATCCGTCGACC |  |
| ChiP-FRT-F | TCGCTGTTCACCTTTGGTGCAGCAATTTATACGTCAAAGAGGATTAACCC GTGTAGGCTGGAGCTGCTTC |  |
| ChiP-FRT-R | GTTGAAGTCAGAACGGTTATCCCACCAGATATCCAGGCGACCGTTTGAGG ATTCCGGGGATCCGTCGACC |  |
| HofQ-FRT-F | TTGCCTGGCAGAATAACAATATCGCCCGCCAGGAGGCGGAGCAGGCGCGG GTGTAGGCTGGAGCTGCTTC |  |
| HofQ-FRT-R | AAATAGATGAAACACGTTCAGCGTAAAAACATTACCGCTTTTACGGCTGT ATTCCGGGGATCCGTCGACC |  |
| NanC-FRT-F | TTTATGCGGGCTTTGTATTGCTTTCTGTATCCTACAAATGAGTGAAATTT GTGTAGGCTGGAGCTGCTTC |  |
| NanC-FRT-R | TGATAGCAAGCGCCGTTATTGTTTTATTCATTGTGACTGTCTCCTGTCTA ATTCCGGGGATCCGTCGACC |  |
| Tsx-FRT-F | ATTTGCGCCTTTTTCACTCCCGCAAGGGATTTTCAAACAGTGGCATACAT GTGTAGGCTGGAGCTGCTTC |  |
| Tsx-FRT-R | ACGTTGTTAACAATCAGAAATGCCGGGAATAAATCCCGGCATTTTCATAA ATTCCGGGGATCCGTCGACC |  |
| UidC-FRT-F | ATCACTAATTAATATTCAATAAAAATAATCAGAACATCAAAGGTGCAACT GTGTAGGCTGGAGCTGCTTC |  |
| UidC-FRT-R | AGTCACAATCGGTGCAAATGTCAGCGTAAATTGCGGCGTTAAACGATAGT ATTCCGGGGATCCGTCGACC |  |
| OmpA-ΔC-F | ACGGCATGCTGAGCCTGGGTGTTTCCTACCGTTTCGGTCAGGGCGAAGCA  GGCAGCGGCGACTACAAAGACGATGACGACAAGtagCTTAGACGTCAGGTGGCACT |  |
| OmpA-ΔC-R | AAAGGCAAAAAAAACCCCGCAGCAGCGGGGTTTTTCTACCAGACGAGAAC ACGCTCAGTGGAACGAAAAC |  |
| OmpA-cfm-F | GAAGGATTTAACCGTGTTATCTCGTTGGAG | Deletion Confirm |
| OmpA-cfm-R | CAGGCATTGCTGGGTAAGGAATAACTGACG |  |
| OmpC-cfm-F | GAGAATGGACTTGCCGACTGATTAATGAGG |  |
| OmpC-cfm-R | CGTGATTATCCTCATGCGAACGGTCGCAAG |  |
| PhoE-cfm-F | CAACGGGACTCTCCCGCTGAATATTCGCGC |  |
| PhoE-cfm-R | GCGGATGATTTTCGTGACGAGCCGGTGAAG |  |
| OmpF-cfm-F | GCAGACACATAAAGACACCAAACTCTCATC |  |
| OmpF-cfm-R | CTGTTTTTGCAAGACGTGAGATTGCTCTGG |  |
| OmpL-cfm-F | GAGATGGCGACAGAAGAAAAAACGGCTTCC |  |
| OmpL-cfm-R | GTGCTTAATCCGGCCAACGAAAACCGTATC |  |
| OmpN-cfm-F | GCGTAACAAATGATATTTATCGGCTAACTG |  |
| OmpN-cfm-R | GATGACACTTTTTGTGTAATGACGGAGTTC |  |
| BglH-cfm-F | TTCGGATGGTATGCAGAGGTGATCAAGACG |  |
| BglH-cfm-R | CCCACTGAGCAGAAATACCGCTGGCAATAG |  |
| CusC-cfm-F | AGCGGGATCAGATGGCAATCGCTTATTGGC |  |
| CusC-cfm-R | GGTCAGGCTTATAGACGGAAAAAATGCCGC |  |
| FadL-cfm-F | AAGATGTACCTCAGACCCTACACTTCGCGC |  |
| FadL-cfm-R | GATACCGGTACGGAAGGTCCAGTTATCATC |  |
| LamB-cfm-F | AAACACACAAAGCCTGTCACAGGTGATGTG |  |
| LamB-cfm-R | TACATACCCACGTACATCATGTCCCAGTTG |  |
| NmpC-cfm-F | TTAGATGAATATTTATCGCGCAGTGACATC |  |
| NmpC-cfm-R | AGGTTGATTTTGTAATCAACGAAAGTAGAC |  |
| PgaA-cfm-F | AGGTGCAAAAATATCTTTCTTTTCAGTTAC |  |
| PgaA-cfm-R | TATAAATGATGTTCTTGACTGGCTAATGCA |  |
| YddB-cfm-F | GAACCTGGCCGATGATATTTGTGACATTAG |  |
| YddB-cfm-R | CTAACGTCAGTAAGAAACAGAGGTTTCTCA |  |
| YfaZ-cfm-F | ATTGAATCCTCTGTGCCAGGCAGTACAATC |  |
| YfaZ-cfm-R | AATCACTATAACGCAAAGGGAGATAAGGCG |  |
| BtuB-cfm-F | CCAACGTCGCATCTGGTTCTCATCATCGCG |  |
| BtuB-cfm-R | GAGATCCGGTAAGAGATGCCGGATCTCGTC |  |
| ChiP-cfm-F | GTTCCTGTAGTCAGCGAGACTTTTCTCAAC |  |
| ChiP-cfm-R | GCCATTATGGCGATGAGAATCAGTTTTTTC |  |
| HofQ-cfm-F | GAAGCAGGCACTACAAACTGTAGTGAAAAG |  |
| HofQ-cfm-R | TCAAATCGGTACTCCTTGTATGCTAAATAC |  |
| NanC-cfm-F | CTACCGGTTGCCAAAGACACTATAAGCCTG |  |
| NanC-cfm-R | GTAATACAGACGCGTTTGCGGCAAATGAAG |  |
| Tsx-cfm-F | GAACTGTGAAACGAAACATATTTTTGTGAG |  |
| Tsx-cfm-R | GCAATATATTGAATTTACGTGCTTTTGTTG |  |
| UidC-cfm-F | GTTGAAATTGATAATCGTAAAAAAGTGCAG |  |
| UidC-cfm-R | GCATAAAAAACGCGCACTTTGTCAACAATC |  |
| pBAD24-LamB-F | CTAGCAGGAGGAATTC ATGATGATTACTCTGCGCAA | pBAD24 |
| pBAD24-LamB-R | GCAGGTCGACTCTAGAG GTTTTGCTATTACCACCAG |  |
| pBAD24-YddB-F | CTAGCAGGAGGAATTC ATGAAGCGAGTTCTTATTCC |  |
| pBAD24-YddB-R | GCAGGTCGACTCTAGA TTACCCGGTTAAGTAAAATC |  |

**Table S3. MICs of various antibiotics for wild-type and porin mutant strains.**

| Strains  Antibiotics | | MG1655 | Δ*ompA* | Δ*ompC* | Δ*phoE* | Δ*ompF* | Δ*ompL* | Δ*ompN* | Δ*bulH* | Δ*nmpC* | Δ*fadL* | Δ*lamB* |
| --- | --- | --- | --- | --- | --- | --- | --- | --- | --- | --- | --- | --- |
| β-Lactam | Ampicillin | 8 | - | - | - | 16 | - | - | - | - | - | - |
|  | Cefalotin | 8 | 4 | - | - | 16 | - | - | - | - | - | - |
|  | Cefoxitin | 2 | - | - | - | 8 | - | - | - | - | - | - |
|  | Ceftazidime | 0.125 | - | - | - | 0.5 | - | - | - | - | - | - |
|  | Aztreonam | 0.03125 | - | - | - | 0.125 | - | - | - | - | - | - |
|  | Imipenem | 0.5 | 0.25 | 0.25 | - | - | - | - | - | - | - | - |
|  | Meropenem | 0.03125 | - | - | - | - | - | - | - | - | - | - |
| Glycopeptide | Vancomycin | 128 | 64 | 64 | - | - | - | - | - | - | - | 256 |
| Metabolite derivative | Fosfomycin | 2 | - | - | - | - | - | - | - | - | - | - |
| Amino acid derivative | D-cycloserine | 8 | - | - | - | 8-16 | - | - | - | - | - | - |
| Aminoglycoside | Streptomycin | 1 | - | 1-2 | - | - | - | - | - | - | - | - |
| Tetracycline | Tetracycline | 0.5 | - | - | - | 1 | - | - | - | - | - | - |
| Amphenicol | Chloramphenicol | 2 | 1 | - | - | 4 | - | - | - | - | - | - |
| Macrolide | Erythromycin | 64 | - | - | - | - | - | - | - | - | - | - |
| Lincosamide | Clindamycin | 128 | 16 | - | - | 256 | - | - | - | - | - | - |
| tRNA derivative | Puromycin | 64 | 32 | 32 | - | - | - | - | - | - | - | - |
| Steroide | Fusidic acid | 512 | - | >1024 | - | >1024 | - | - | - | - | - | - |
| Polyketide | Mupirocin | 256 | 128 | - | - | - | - | - | - | - | - | - |
| Quinolone | Nalidixic acid | 4 | - | - | - | - | - | - | - | - | - | - |
|  | Cinoxacin | 2 | - | - | - | 4 | - | - | - | - | - | - |
| Aminocoumarin | Novobiocin | 64 | - | - | - | - | - | - | - | - | - | - |
| Anti-folate | Trimethoprim | 0.25 | 0.125 | - | - | - | - | - | - | - | - | - |
| Ansamycin | Rifampicin | 6 | 5 | - | - | - | - | - | - | - | 7 | 8 |
| Peptide | Colistin | 0.5 | - | - | - | - | - | - | - | - | - | - |
| Etc. | Nitrofurantoin | 8 | - | 16 | - | - | - | - | - | - | - | - |

| Strains  Antibiotics | | MG1655 | Δ*pgaA* | Δ*yddB* | Δ*yfaZ* | Δ*btuB* | Δ*chiP* | Δ*hopQ* | Δ*nanC* | Δ*tsx* | Δ*uidC* |
| --- | --- | --- | --- | --- | --- | --- | --- | --- | --- | --- | --- |
| β-lactam | Ampicillin | 8 | - | - | - | - | - | - | - | - | - |
|  | Cefalotin | 8 | - | - | - | - | - | - | - | - | - |
|  | Cefoxitin | 2 | - | - | - | - | - | - | - | - | - |
|  | Ceftazidime | 0.125 | - | - | - | - | - | - | - | - | - |
|  | Aztreonam | 0.03125 | - | - | - | - | - | - | - | - | - |
|  | Imipenem | 0.5 | - | - | - | - | - | - | - | - | - |
|  | Meropenem | 0.03125 | - | - | - | - | - | - | - | - | - |
| Glycopeptide | Vancomycin | 128 | - | - | - | - | - | - | - | - | - |
| Metabolite derivative | Fosfomycin | 2 | - | - | - | - | - | - | - | - | - |
| Amino acid derivative | D-cycloserine | 8 | - | - | - | - | - | - | - | - | 8-16 |
| Aminoglycoside | Streptomycin | 1 | 2-4 | 1-2 | - | - | 1-2 | - | - | 2 | 2-4 |
| Tetracycline | Tetracycline | 0.5 | - | - | - | - | - | - | - | - | - |
| Amphenicol | Chloramphenicol | 2 | - | - | - | - | - | - | - | - | - |
| Macrolide | Erythromycin | 64 | - | - | - | - | - | - | - | - | - |
| Lincosamide | Clindamycin | 128 | - | - | - | - | - | - | - | - | - |
| tRNA derivative | Puromycin | 64 | - | - | - | - | - | - | - | - | - |
| Steroide | Fusidic acid | 512 | - | - | - | - | - | - | - | - | - |
| Polyketide | Mupirocin | 256 | - | - | - | - | - | - | - | - | - |
| Quinolone | Nalidixic acid | 4 | - | - | - | - | - | - | - | - | - |
|  | Cinoxacin | 2 | - | - | - | - | - | - | - | - | - |
| Aminocoumarin | Novobiocin | 64 | - | 512 | - | - | - | - | - | - | - |
| Anti-folate | Trimethoprim | 0.25 | - | - | - | - | - | - | - | - | - |
| Ansamycin | Rifampicin | 6 | - | - | - | - | - | - | - | - | - |
| Peptide | Colistin | 0.5 | - | - | - | - | - | - | - | - | - |
| Etc. | Nitrofurantoin | 8 | - | - | - | - | - | - | - | - | - |

**References**

Blattner, F. R., Plunkett, G., 3rd, Bloch, C. A.*, et al.* (1997). The complete genome sequence of *Escherichia coli* K-12. *Science*. 277, 1453-1462.

Datsenko, K. A., Wanner, B. L. (2000). One-step inactivation of chromosomal genes in *Escherichia coli* K-12 using PCR products. *Proc. Natl. Acad. Sci. USA*. 97, 6640-6645. doi: 10.1073/pnas.120163297
